# Acute effects of cigarette smoking on inflammation in healthy intermittent smokers

**DOI:** 10.1186/1465-9921-6-22

**Published:** 2005-03-01

**Authors:** Hester van der Vaart, Dirkje S Postma, Wim Timens, Machteld N Hylkema, Brigitte WM Willemse, H Marike Boezen, Judith M Vonk, Dorothea M de Reus, Henk F Kauffman, Nick HT ten Hacken

**Affiliations:** 1 Department of Pulmonology, University Medical Center Groningen, Groningen, the Netherlands; 2 Department of Pathology, University Medical Center Groningen, Groningen, the Netherlands; 3 Department of Allergology University Medical Center Groningen, Groningen, the Netherlands; 4 Department of Epidemiology and Statistics, University Medical Center Groningen, Groningen, the Netherlands

**Keywords:** Sputum, Chronic Obstructive Pulmonary Disease, Inflammation, Tobacco, Carbon Monoxide

## Abstract

**Background:**

Chronic smoking is the main risk factor for chronic obstructive pulmonary disease. Knowledge on the response to the initial smoke exposures might enhance the understanding of changes due to chronic smoking, since repetitive acute smoke effects may cumulate and lead to irreversible lung damage.

**Methods:**

We investigated acute effects of smoking on inflammation in 16 healthy intermittent smokers in an open randomised cross-over study. We compared effects of smoking of two cigarettes on inflammatory markers in exhaled air, induced sputum, blood and urine at 0, 1, 3, 6, 12, 24, 48, 96 and 192 hours and outcomes without smoking. All sputum and blood parameters were log transformed and analysed using a linear mixed effect model.

**Results:**

Significant findings were: Smoking increased exhaled carbon monoxide between 0 and 1 hour, and induced a greater decrease in blood eosinophils and sputum lymphocytes between 0 and 3 hours compared to non-smoking. Compared to non-smoking, smoking induced a greater interleukin-8 release from stimulated blood cells between 0 and 3 hours, and a greater increase in sputum lymphocytes and neutrophils between 3 and 12 hours.

**Conclusion:**

We conclude that besides an increase in inflammation, as known from chronic smoking, there is also a suppressive effect of smoking two cigarettes on particular inflammatory parameters.

## Background

Chronic obstructive pulmonary disease (COPD) is one of the leading causes of morbidity and mortality world-wide, and its prevalence is still rising [1]. In order to develop strategies for its prevention and treatment, it is important to understand the underlying pathophysiologic mechanisms of this disease. Since chronic smoking is the main risk factor to develop COPD most studies in this field have been carried out in chronic (ex)smokers with or without COPD. It is also important to study the initial response to cigarette smoke to better understand the effects of chronic smoking, since repetitive acute smoke effects may cumulate and ultimately lead to irreversible lung damage associated with COPD. In addition, to appropriately evaluate the impact of chronic smoking, the "background" effects of acute smoking should be determined.

Until now, only a few studies have investigated acute effects of smoking in humans [2]. Unfortunately, these studies investigated only a small number of time points after smoking, hence little information is available on the time course and resolution of smoking induced changes. Furthermore, all studies assessed acute effects of smoking in chronic smokers who refrained from smoking for maximally 24 hours. It is unknown whether this is sufficiently long to exclude the influence of previous smoking on the acute smoke results. Finally, no study so far investigated acute smoke effects in sputum.

In the present study we investigated acute effects of smoking of two cigarettes by healthy intermittent smokers who refrained from smoking nine days before the study period. In this way, temporary effects on the airways due to chronic smoking will probably not affect the acute response to smoke. We assessed the time effects of cigarette smoking on both induction and resolution of the inflammatory response in exhaled air, induced sputum, blood and urine. We hypothesised that smoking of two cigarettes would induce an increase in inflammatory cells and markers within a limited time interval.

## Methods

### Design of the study

We performed a randomised, two-period cross-over, pilot study. Subjects were randomised into smoking two cigarettes or no smoking. Subjects refrained from smoking during nine days before each study period, verified by exhaled carbon monoxide (CO < 6 ppm) and urinary cotinine (< 25 ng/ml). The time interval between the two study periods varied between 9 to 20 days. Measurements of exhaled CO, exhaled Nitric Oxide (NO), blood sampling and Forced Expiratory Volume in 1 second (FEV_1_) were performed immediately before (baseline) and 1, 3, 6, 12, 24, 48, 96 and 192 hours after smoking and at the same time points in the no smoking period. Sputum was induced at 3, 6, 12, 24, 48, 96, 192 hours after smoking and no smoking. All subjects smoked two cigarettes from the same brand within 30 minutes and were encouraged to inhale deeply (Caballero unfiltered cigarettes, tar 12 mg, nicotine 1.0 mg, commercially obtained, no gifts). Adequacy of smoke inhalation was verified by the investigator. The working groups sputum induction from the ERS stated recently that sputum inductions should not be repeated within 48 hours to avoid *carry over *effects [3]. Taking this into account, we used a cross-over design (including no smoking) in this study to correct for this *carry over *effect. We have analysed the results of the control arm as a separate study in order to investigate the induction and resolution of the inflammatory response generated by repeated sputum inductions [4].

### Subjects

Sixteen healthy intermittent smokers were recruited by advertisements in the local newspaper. Intermittent smoking was defined as smoking more than one cigarette a month, but not daily, during the last 6 months. We chose to investigate intermittent smokers because they are able to refrain from smoking for a certain time period (in contrast to most current smokers) and they are used to inhale smoke (in contrast to non-smokers). Included were subjects older than 40 years, with normal lung function (prebronchodilator FEV_1_/IVC [Inspiratory Vital Capacity] > 89% of predicted for women and > 88% of predicted for men [5] and a prebronchodilator FEV_1 _> 1 litre). Excluded were subjects with: 1) a history of asthma, allergic rhinitis, or allergic eczema; 2) atopy, confirmed by a positive skin prick test; 3) any current respiratory disease, symptoms of cough or sputum production; 4) a respiratory tract infection within the preceding 8 weeks or a nasal infection within the preceding 4 weeks; 5) treatment with glucocorticosteroids within the preceding 8 weeks; 6) use of aspirin, NSAIDs, paracetamol or antihistamines within the preceding 4 weeks. Subjects were asked to avoid places with high environmental tobacco smoke exposure during the study periods. The study was approved by the medical ethics committee of the University Medical Center Groningen, the Netherlands. Written informed consent was obtained from all subjects.

### Pulmonary function, exhaled NO and CO

FEV_1 _and IVC were measured according to the guidelines of the European Respiratory Society [5], using a pneumotachograph (Jaeger, Wurzberg, Germany). Exhaled NO levels were determined according to the guidelines of the American Thoracic Society [6], exhaling with a flow of 100 ml/sec against a resistance between 5 and 20 cm H_2_O, using a chemiluminescence analyser (Ecophysics CLD 700 AL). Exhaled CO levels were measured using an infrared CO analyser (UNOR 6N, Maihak AG, Hamburg, Germany) [7].

### Blood analyses

Blood differential cell counts were analysed automatically with a haematology flow cytometer (Coulter-STKS, Beckman Coulter, Miami, USA). Flow cytometric analysis was performed on blood cells using peridinin chlorophyll protein (PerCP) labelled anti-human leukocyte antigen (HLA)-DR, phycoerythrin (PE) labelled anti-CD11b, allophycocyanin (APC) labelled CD14 and fluorescein-isothiocyanate (FITC) labelled CD63 monoclonal antibodies (Becton Dickinson, Franklin Lakes, NJ USA). HLA-DR, CD63 and CD11b are activation markers for respectively monocytes and granulocytes. CD14 is used to discern between monocytes and granulocytes. Functional assays were performed on unstimulated and lipopolysacharide (LPS, 1 ng/ml, BioWhittaker, Walkerville, USA) stimulated blood cells, measuring tumor necrosis factor (TNF)-α, interleukin (IL)-1β, IL-8 and IL-10 by ELISA (Sanquin, Amsterdam, the Netherlands).

### Sputum induction and processing

Sputum was induced according to a modified standard technique [8], using 4.5% hypertonic saline. Whole sputum was processed within 120 minutes according to the modified method of Rutgers and colleagues [8]. The cell-free supernatant was collected and stored in aliquots at -80°C pending analysis of soluble mediators.

### Sputum analyses

Flow cytometric analysis was performed on sputum cells using PerCP labelled anti-HLA-Dr, PE labelled anti-CD11b, APC labelled CD14 and FITC labelled CD63 monoclonal antibodies (Becton Dickinson, Franklin Lakes, NJ USA). Immunocytology was performed to quantify the percentage of inducible NO synthase (iNOS) positive macrophages. Cytospins were double stained with a monoclonal antibody against CD68 (IgG1 isotype, Dako, Glostrup, Danmark) as a marker for macrophages and rabbit polyclonal antiserum against iNOS (Transduction Laboratories, Lexington, KY, USA).

The following soluble mediators were measured in sputum supernatant. NO_2_^-^/NO_3_^- ^was measured using the Griess reaction, eosinophilic cationic protein (ECP) using the fluorenzyme immunoassay UniCAP ECP (Pharmacia, Uppsala, Sweden). IL-8 and Leukotriene B4 (LTB4) were measured by a commercial ELISA (IL-8: Sanquin, Amsterdam, the Netherlands, LTB4: Amersham Biosciences, UK). Matrix metalloproteinase-9 (MMP-9) was measured by gelatine zymography [9], and tissue inhibitor of metalloproteinase-1 (TIMP-1) by ELISA (R&D, Abingdon, UK). Neutrophil elastase (NE) activity was measured by chromogenic substrate assay (N-methoxysuccinyl-ala-ala-pro-val-p-nitoanilide, Sigma, UK)] [10].

### Urinary measurements

Before inhalation of smoke (or control), a urine portion was collected to measure urine cotinine. Cotinine was measured by gaschromatography-mass-spectrometry (Pharmacy Department, Groningen, the Netherlands). Furthermore, urine was collected over 24 hours in five consecutive fractions: 0–1 hour, 1–3 hours, 3–6 hours, 6–12 hours and 12–24 hours from all subjects to assess leukotriene E4 levels (ELISA, Amersham Biosciences, UK).

### Statistical analyses

Since the start and duration of the acute effects of smoking of two cigarettes on our parameters were unknown, time series of all variables were plotted. Based on visual inspection of these plots the time intervals to be analysed were selected. The slopes of parameters were estimated using linear mixed effect models [11] by including the variables time (hours), smoking (yes or no) and their interaction. For the sputum parameters no baseline values were present, therefore time point 192 hours was used as baseline value. After log-transformation of all blood and sputum variables, the residuals of the models were normally distributed. All analyses were performed in S-plus 2000 (Insightful Corporation, Seattle, WA, USA). A p value <0.05 was considered statistically significant.

## Results

### Subjects

Clinical characteristics of the 16 subjects are listed in table 1. Fifteen subjects successfully refrained from smoking for nine days. One subject smoked one cigarette five days before the start of the study, but the urinary cotinine and exhaled CO levels were within the required range. The analyses are performed on data from all 16 subjects.

**Table 1:** Subject characteristics (healthy intermittent smokers).

Sex, male/female	12/4
Age, years	49 (39–71)
Smoked pack years	4 (0–40)
Smoked cigarettes per month	14 (1–60)
FEV_1_, % predicted	119 (68–144)
FEV_1_/ IVC, %	77.6 (68.1–87.0)

Values expressed as medians (ranges). FEV_1_: Forced Expiratory Volume in 1 second, IVC: Inspiratory Vital Capacity.

### Exhaled NO and CO and FEV_1_

**Figure 1:**
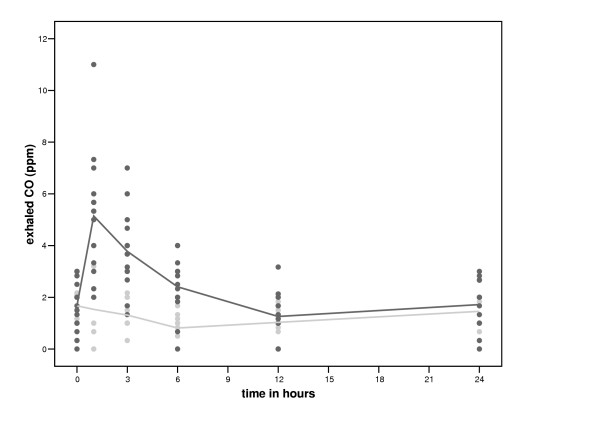
**Time course of smoking of two cigarettes on exhaled carbon monoxide (CO). **Black circles represent the values after smoking two cigarettes and grey circles represent the values of the control period.

Exhaled CO increased significantly more with smoking than without between 0 and 1 hour and subsequently decreased significantly more between 1 and 12 hours (table 2, figure 1). Smoking had no significant effect on exhaled NO (data not shown) or FEV_1 _(table 2).

**Table 2:** Linear mixed effect models: CO and FEV_1_

**Independent variable**	**Time interval**	**B**	**95% CI**	**P value**
CO, ppm	0–1 hour	3.61	2.67–4.54	**<0.0001**
	1–12 hours	-0.29	-0.38 – -0.21	**<0.0001**

FEV_1_, L/sec	0–1 hour	0.06	-0.07–0.20	0.38

The time intervals of the above parameters were selected based on visual inspection of the plots. The slopes of the parameters were estimated using linear mixed effect models [11] by including the variables time (hours), smoking (yes or no) and their interaction. B: regression coefficient for the variable time (for further information see the method section). CO: carbon monoxide; FEV_1_: Forced Expiratory Volume in 1 second.

### Blood

**Figure 2:**
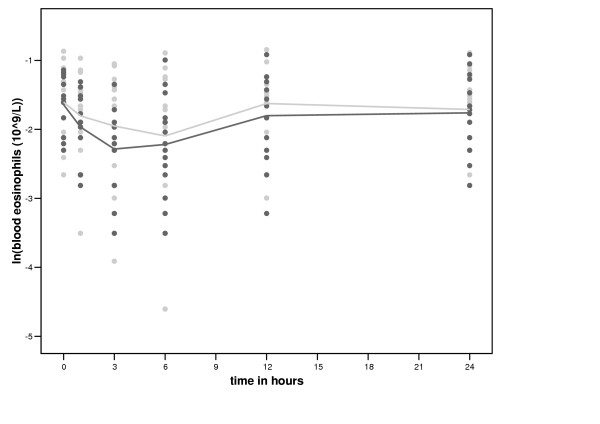
**Time course of smoking of two cigarettes on blood eosinophils. **Black circles represent the values after smoking two cigarettes and grey circles represent the values of the control period.

The number of blood eosinophils decreased more with smoking than without between 0 and 3 hours (table 3 and figure 2). Smoking had no significant effect on the number of other blood cells (table 3 and additional file 1, table 1). IL-8 release from LPS stimulated blood cells increased more with smoking than without between 0 and 3 hours (table 3). Smoking had no significant effect on TNF-α, IL-10 and IL-1β release compared with no smoking (additional file 1, table 2). There was no significant difference in the expression of CD11b, CD63 and HLA-DR on CD14 high and CD14 low cells between smoking and no smoking (data not shown).

**Table 3:** Linear mixed effect models: blood cells, IL-8 and TNF-α

**Independent variable**	**Time interval**	**B**	**95% CI**	**P value**
Log (leucocytes, 10^9^/L)	0–12 hours	0.00	-0.01–0.01	0.44
Log (neutrophils, 10^9^/L)	0–12 hours	0.00	-0.01–0.02	0.58
Log (monocytes, 10^9^/L)	0–1 hour	0.04	-0.10–0.19	0.55
Log (eosinophils, 10^9^/L)	0– 3 hours	-0.11	-0.18 – -0.03	**0.01**
Log (lymphocytes, 10^9^/L)	0–12 hours	-0.00	-0.01–0.01	0.65
Log (IL-8, pg/ml)*	0–3 hours	0.09	0.04–0.14	**0.001**
Log (TNF-α, pg/ml)**	0–3 hours	0.02	-0.08–0.12	0.75

The time intervals of the above parameters were selected based on visual inspection of the plots. The slopes of the parameters were estimated using linear mixed effect models [11] by including the variables time (hours), smoking (yes or no) and their interaction. B: regression coefficient for the variable time (for further information see the method section). * Release from whole blood cells after lipopolysacharide (LPS) stimulation. ** Spontaneous release from whole blood cells IL: interleukin, TNF-α: tumor necrosis factor-α

### Sputum

**Figure 3:**
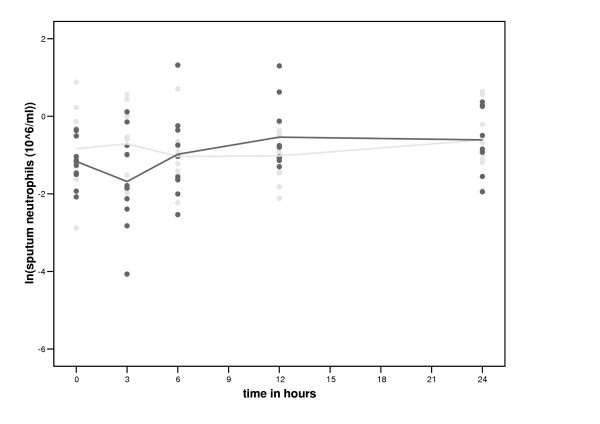
**Time course of smoking of two cigarettes on sputum neutrophils. **Black circles represent the values after smoking two cigarettes and grey circles represent the values of the control period.

**Figure 4:**
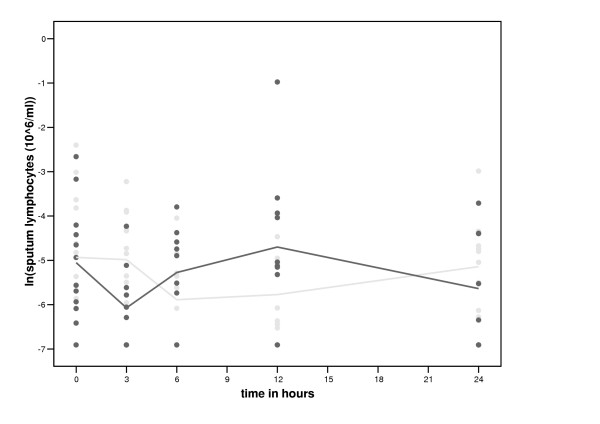
**Time course of smoking of two cigarettes on sputum lymphocytes. **Black circles represent the values after smoking two cigarettes and grey circles represent the values of the control period.

**Figure 5:**
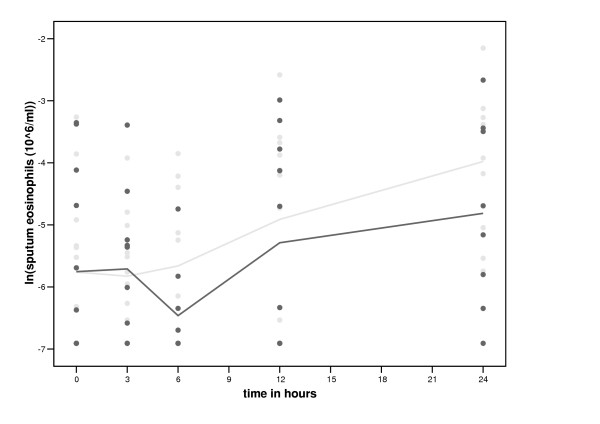
**Time course of smoking of two cigarettes on sputum eosinophils. **Black circles represent the values after smoking two cigarettes and grey circles represent the values of the control period.

The total number and percentage of sputum cells within the first 24 hours after smoking and no smoking are shown in table 4. The number of neutrophils increased significantly more with smoking than without between 3 and 12 hours (table 5, figure 3). The number of sputum lymphocytes decreased more with smoking than without between 0 and 3 hours (table 5, figure 4). Subsequently, however, the percentage and number of sputum lymphocytes increased more with smoking than without between 3 and 12 hours (table 5, figure 4). Smoking had no significant effect on the percentage and number of sputum eosinophils (table 5, figure 5) and macrophages (table 5). Smoking had also no significant effect on the levels of inflammatory mediators in sputum (additional file 1, table 3) and the expression of CD11b, CD63 and HLA-DR on CD14 high and CD14 low cells and the number of iNOS positive macrophages (data not shown).

**Table 4:** Inflammatory cells in sputum after smoking and no smoking.

	**Baseline (192 hours)**	**3 hours**	**6 hours**	**12 hours**	**24 hours**
**SMOKING**					

Sputum cells, 10^6^/ml	1.8 (0.1–16.3)	2.0 (0.3–6.6)	2.4 (0.1–7.3)	2.4 (0.5–6.6)	2.6 (0.0–9.0)
Neutrophils, %	56.9 (22.0–97.3)	56.4 (4.0–96.0)	83.2 (13.7–97.3)	77.5 (32.2–98.3)	67.3 (39.0–84.3)
Macrophages, %	37.9 (2.5–74.5)	42.2 (3.7–84.8)	13.8 (2.5–68.5)	16.8 (1.7–61.7)	27.4 (14.8–57.7)
Eosinophils, %	0.1 (0.0–6.2)	0.5 (0.0–5.2)	0.0 (0.0–0.3)	1.1 (0.0–8.3)	1.0 (0.0–5.2)
Lymphocytes, %	1.1 (0.0–3.8)	0.4 (0.0–1.7)	1.0 (0.0–2.0)	1.2 (0.0–7.3)	0.4 (0.0–1.8)

**NO SMOKING**					

Sputum cells, 10^6^/ml	2.8 (0.8–23.8)	3.1 (0.1–20.4)	2.0 (0.7–7.9)	2.1 (0.4–6.2)	2.1 (0.6–9.5)
Neutrophils, %	50.9 (20.3–84.8)	58.9 (31.8–94.2)	73.2 (22.8–94.7)	83.2 (26.7–98.3)	64.5 (29.0–80.3)
Macrophages, %	46.9 (15.0–77.7)	38.5 (4.2–64.0)	20.8 (4.5–71.2)	10.3 (1.7–67.8)	28.7 (16.0–66.5)
Eosinophils, %	0.2 (0.0–3.2)	0.3 (0.0–1.2)	0.2 (0.0–4.2)	1.7 (0.0–15.5)	2.2 (0.5–12.5)
Lymphocytes, %	0.7 (0.0–4.0)	0.9 (0.0–2.8)	0.4 (0.0–3.5)	0.2 (0.0–3.7)	0.9 (0.0–1.5)

Values are expressed as medians (ranges).

**Table 5:** Linear mixed effect models of sputum cells

**Independent variable**	**Time interval**	**B**	**95% CI**	**P value**
Log (total cells, 10^6^/ml)	0–3 hours	0.075	-0.15–0.30	0.52

Log (neutrophils, % and 10^6^/ml)				
%	0–3 hours	-0.17	-0.34 – -0.00	0.07
number	0–3 hours	-0.22	-0.49–0.30	0.11
%	3–12 hours	0.03	-0.02–0.07	0.27
number	3–12 hours	0.13	0.04–0.22	**0.007**

Log (macrophages, % and 10^6^/ml)				
%	0–3 hours	0.12	-0.01–0.25	0.08
number	0–3 hours	0.13	-0.11–0.36	0.31

Log (eosinophils, % and 10^6^/ml)				
%	0–6 hours	-0.22	-0.71–028	0.39
number	0–6 hours	-0.12	-0.30–0.06	0.20
%	3–6 hours	-0.84	-3.63–1.96	0.57
number	3–6 hours	-0.31	-1.35–0.73	0.57

Log (lymphocytes, % and 10^6^/ml)				
%	0–3 hours	-0.28	-0.59–0.02	0.08
number	0–3 hours	-0.26	-0.42–-0.11	**0.004**
%	3–12 hours	0.19	0.06–0.31	**0.006**
number	3–12 hours	0.23	0.09–0.36	**0.002**

The time intervals of the above parameters were selected based on visual inspection of the plots. The slopes of the parameters were estimated using linear mixed effect models [11] by including the variables time (hours), smoking (yes or no) and their interaction. B: regression coefficient for the variable time (for further information see the method section).

### Urine

Smoking had no significant effect on leukotriene E4 levels in urine compared to no smoking (data not shown).

## Discussion

In order to better understand the effects of chronic smoking, it is important to study the initial (acute) response to cigarette smoke, since repetitive acute smoke effects may cumulate and ultimately lead to irreversible damage. We therefore investigated the acute effects of cigarette smoking on both induction and resolution of the inflammatory response in healthy intermittent smokers. This study shows that smoking of two cigarettes acutely suppresses blood eosinophils. Furthermore, smoking induces a biphasic response in sputum lymphocytes, after an initial smoke-related suppression, the cells increase more with smoking than without. Finally, smoking increases sputum neutrophils and the release of IL-8 from whole blood cells.

A remarkable finding in our study is that smoking of two cigarettes decreases eosinophils in blood. Three other studies have reported similar results: eosinophils decreased in blood from healthy female smokers within two hours after smoking 12 cigarettes [12], in lung tissue of rats within 6 hours after smoke exposure [13], and in lung lavage fluid of ovalbumin sensitised mice after 3 weeks smoke exposure [14]. A decrease in eosinophils may be due to a direct (apoptotic) effect by toxic substances in cigarette smoke [15], or to anti-inflammatory substances in cigarette smoke, like CO [16,17]. Smoking did not show a significant suppressive effect on sputum eosinophils in our study, although the figures show that sputum eosinophils are decreasing more from 3 hours onwards with smoking than without. The reason for this is probably a lack of study power, due to the lower number of successful measurements in sputum than in blood, or due to the low baseline levels of sputum eosinophils in our healthy non-atopic subjects. One has to realise that the decrease in eosinophils in blood in our study is significant but relatively small.

This study is the first to report that sputum can be used to study acute smoke effects. The number of sputum neutrophils increased between 3 and 12 hours after smoking. In line with this, we demonstrated a higher release of IL-8 by LPS stimulated blood cells after smoking, which may have contributed to increased neutrophil chemotaxis. The rise in neutrophils is in line with two studies on acute effects of smoking in humans, showing increased neutrophils in bronchoalveolar lavage fluid 1 hour after smoking [18] and increased neutrophil retention in the lung during smoke exposure [19]. The fast increase in neutrophils in sputum might result from detachment of neutrophils from the pulmonary vascular endothelium (the so-called marginated pool) [20] or from recruitment from the bone marrow [21,22].

Smoking also shortly suppressed the number of lymphocytes in sputum. Thereafter sputum lymphocytes increased more with smoking than without. The initial decrease might result from increased adherence of lymphocytes in the lung tissue due to the fast upregulation of adhesion molecules after smoking [23] or may also be caused by the suppressive CO as mentioned in the prior paragraph [16]. The subsequent increase in sputum lymphocytes may reflect the outwash of lymphocytes from the tissue into the sputum, which can be regarded as the waste bin of lung inflammatory cells.

Smoking did not affect all inflammatory markers we investigated. A few factors may contribute to this lack of response. First, the number of subjects and the number of cigarettes (n = 2) might have been too low. Second, we may have included a heterogeneous group of subjects regarding their response to cigarette smoke. We know that approximately 80% of all smokers never develop COPD. Therefore it is conceivable that a part of our healthy smokers does not respond to cigarette smoking. Third, we included subjects with a broad range in current and past smoking. Fourth, sputum may reflect only a part of the acute inflammatory changes of the airway wall [8]. It would be interesting to study the acute effects of smoking on lung tissue. Finally, CO in cigarette smoke may have dampened the inflammatory response, especially in the early phase. After continuous smoking the damaging and irritating effects may prevail, giving rise to more pronounced inflammation.

Studying the acute effects of smoking in intermittent healthy smokers has both advantages and disadvantages. We choose the presented model for a number of reasons. First, intermittent smokers can refrain from smoking for three weeks in contrast to most current smokers. Second, intermittent smokers have a normal lung function (in contrast to COPD), and likely (nearly) no structural airway changes, which may affect a normal response to cigarette smoke. Third, we assumed that detecting an acute inflammatory response to cigarette smoking after an abstinence period of 9 days would be easier than detecting a response on top of chronic smoke exposure. Finally, intermittent smokers are used to inhale cigarette smoke (in contrast to non-smokers). We realise that our model has the disadvantage that the results of our study cannot easily be extrapolated to the chronic effects of smoking or COPD development. Nevertheless, when comparing the airway inflammation of our subjects with that of smoking COPD patients, both show increased levels of neutrophils, lymphocytes and IL-8 in sputum. However, in COPD patients after quitting smoking lymphocytes and neutrophils do not normalise [24], in contrast to the short-lived acute effects of smoking in this study. This suggests that not smoke but structural changes in the airways are responsible for the ongoing inflammation in COPD. Despite above limitations we think that knowledge on both the acute and chronic effects of smoking will help to better understand the mechanisms of cigarette smoke induced inflammation, which may underlie the development of COPD.

## Conclusion

We conclude that besides an increase in inflammation, as known from chronic smoking, there is also a suppressive effect of smoking of two cigarettes on particular inflammatory parameters. Although this seems beneficial, it may disturb physiologic responses, like repair processes, in which inflammatory cells play a role.

## Competing interests

The author(s) declare that they have no competing interests.

## Authors' contributions

HV: Participated in the design of the study, performed the study and drafted the manuscript.

DP: Conceived the study, participated in the design and co-ordination of the study and helped draft the manuscript.

WT: Participated in the design of the study and helped draft the manuscript.

MH: Participated in the design of the study, co-ordinated the FACS analyses and helped draft the manuscript.

BW: Performed some of the laboratory analyses, participated in the design of the study and helped draft the manuscript.

HB: Performed statistical analyses and helped draft the manuscript.

JV: Performed statistical analyses and helped draft the manuscript.

DR: Performed and co-ordinated most laboratory analyses and helped draft the manuscript.

HK: Co-ordinated laboratory analyses, participated in the design of the study and helped draft the manuscript.

NH: Conceived the study, participated in the design and co-ordination of the study and helped draft the manuscript.

## Supplementary Material

Additional File 1 Table 1. Number of blood cells (10^9^/L) after smoking and no smoking. Table 2. Release of IL-1β, IL-10, IL-8 and TNF-α from blood cells after smoking and no smoking. Table 3. Inflammatory mediators in sputum after smoking and no smoking. Click here for file
