# Supplementary material for: Acute effects of cigarette smoking on inflammation in healthy intermittent smokers
Source: Respir Res. 2005 Mar 1;6(1):22. doi: 10.1186/1465-9921-6-22 (PMC554761; doi:10.1186/1465-9921-6-22)
Supplement: Additional File 1 — Table 1. Number of blood cells (109/L) after smoking and no smoking. Table 2. Release of IL-1β, IL-10, IL-8 and TNF-α from blood cells after smoking and no smoking. Table 3. Inflammatory mediators in sputum after smoking and no smoking. [file 1465-9921-6-22-S1.doc]

# Addional FILE 1

Acute effects of cigarette smoking on inflammation in healthy intermittent smokers.

Hester van der Vaart^1^, Dirkje S. Postma^1^, Wim Timens^2^, Henk F. Kauffman^3^, Machteld N. Hylkema^2^, Brigitte W.M. Willemse^2^, H. Marike Boezen^4^, J.M.Vonk^4^, Dorothea M. de Reus^3^, Nick H.T. ten Hacken^1^

Department of Pulmonology^1^, University Medical Center Groningen, Groningen, the Netherlands

Department of Pathology^2^, University Medical Center Groningen, Groningen, the Netherlands

Department of Allergology^3^ University Medical Center Groningen, Groningen, the Netherlands

# Department of Epidemiology and Statistics^4^, University Medical Center Groningen, Groningen, the Netherlands

Table 1. Number of blood cells (10^9^/L) after smoking and no smoking.

|  | 0 hours | 1 hour | 3 hours | 6 hours | 12 hours | 24 hours |
| --- | --- | --- | --- | --- | --- | --- |
| SMOKING | | | | | | |
| Leukocytes | 5.5 (4.0-7.6) | 6.0 (3.9-8.1) | 6.4 (4.6-9.9) | 7.3 (4.4-12.9) | 7.1 (4.4-11.9) | 5.5 (4.1-8.6) |
| Neutrophils | 2.9 (1.6-5.0) | 3.6 (1.9-5.4) | 4.0 (2.8-6.7) | 4.0 (2.6-10.2) | 4.1 (2.3 8.3) | 3.2 (1.9-6.0) |
| Lymphocytes | 1.7 (1.2-2.4) | 1.7 (1.1-2.5) | 1.9 (1.0-2.8) | 1.8 (1.0-3.3) | 2.3 (1.4-3.7) | 1.7 (1.1-2.7) |
| Monocytes | 0.5 (0.4-0.7) | 0.5 (0.4-0.7) | 0.4 (0.3-0.7) | 0.5 (0.3-0.8) | 0.6 (0.4-0.9) | 0.5 (0.3-0.6) |
| Eosinophils | 0.2 (0.1-0.3) | 0.2 (0.1-0.3) | 0.1 (0.0-0.3) | 0.1 (0.0-0.4) | 0.2 (0.0-0.4) | 0.2 (0.1-1.1) |
| NO SMOKING | | | | | | |
| Leukocytes | 5.6 (3.2-9.4) | 6.4 (3.6-8.3) | 6.2 (3.7-8.2) | 7.1 (3.8-10.2) | 7.6 (4.7-10.40 | 5.7 (3.4-8.0) |
| Neutrophils | 3.0 (1.8-6.7) | 3.8 (2.1-5.6) | 3.8 (1.9-5.3) | 4.1 (2.0-1.9) | 4.2 (2.2-6.8) | 3.3 (1.8-5.8) |
| Lymphocytes | 1.7 (0.8-2.6) | 1.9 (1.0-2.5) | 1.9 (1.0-3.0) | 2.0 (1.3-3.2) | 2.4 (1.6-3.7) | 1.6 (1.2-2.5) |
| Monocytes | 0.5 (0.4-0.7) | 0.5 (0.3-0.6) | 0.5 (0.3-0.7) | 0.5 (0.4-0.8) | 20.5 (0.4-0.9) | 0.5 (0.2-0.7) |
| Eosinophils | 0.3 (0.1-0.4) | 0.2 (0.0-0.4) | 0.2 (0.0-0.4) | 0.2 (0.0-0.4) | 0.2 (0.1-0.4) | 0.2 (0.1-0.4) |

Values are expressed as medians (ranges).

Table 2. Release of IL-1β, IL-10, IL-8 and TNF-α from blood cells after smoking and no smoking.

|  | 0 hours | 3 hours | 24 hours |
| --- | --- | --- | --- |
| SMOKING | | | |
| IL-1β | 15.0 (0.9-133) | 13.9 (1.6-112) | 19.3 (1.1-274) |
| LPS induced IL-1β | 1435 (752-2970) | 2400 (474-4784) | 2392 (723-4020) |
| IL-10 | 2.3 (0.3-73.4) | 2.9 (0.3-62.1) | 2.3 (0.2-97.7) |
| LPS induced IL-10 | 22.6 (5.1-131) | 32.5 (5.5-161) | 38.7 (13.7-183) |
| IL-8 | 328 (22.8-1710) | 343 (25.3-2893) | 352 (11.5-2457) |
| LPS induced IL-8 | 4066 (1276-7714) | 6031 (1844-13162) | 6736 (2284-13533) |
| TNF-α | 10.3 (2.6-39.9) | 10.0 (1.3-35.8) | 14.0 (2.1-63.3) |
| LPS induced TNF-α | 2011 (564-4470) | 2323 (516-4298) | 1957 (827-4701) |
| NO SMOKING | | | |
| IL-1β | 29.3 (1.7-127) | 13.0 (3.7-127) | 15.6 (2.6-148) |
| LPS induced IL-1β | 1993 (480-5569) | 2387 (471-6979) | 1746 (571-4439) |
| IL-10 | 1.7 (0.3-75.8) | 2.9 (0.6-70.9) | 1.4 (0.2-78.4) |
| LPS induced IL-10 | 24.9 (7.8-131) | 22.3 (7.1-164) | 32.8 (7.4-141) |
| IL-8 | 192 (15.8-1537) | 266 (71.0-1730) | 287 (84.2-1082) |
| LPS induced IL-8 | 4631 (869-12113) | 5368 (1099-10328) | 4592 (2268-17671) |
| TNF-α | 12.2 (3.7-68.6) | 10.4 (5.2-41.2) | 12.4 (1.7-33.1) |
| LPS induced TNF-α | 2035 (749-6822) | 2021 (917-5487) | 2167 (636-5705) |

Spontaneous and LPS (1 ng/ml) stimulated release of IL-1β, IL-10, IL-8 and TNF-α (pg/ml) from whole

blood cells after smoking and no smoking. Values are expressed as medians (ranges). IL: interleukin, LPS: lipopolysacharide, TNF-α: tumor necrosis factor-α.

Table 3. Inflammatory mediators in sputum after smoking and no smoking.

|  | Baseline  (192 hours) | 3 hours | 6 hours | 12 hours | 24 hours |
| --- | --- | --- | --- | --- | --- |
| SMOKING | | | | | |
| IL-8, pg/ml | 1813 (590-16230) | 1635 (539-6392) | 1152 (420-4576) | 2034 (596-6134) | 1591 (426-13180) |
| ECP, ng/ml | 77.9 (12.9-264) | 37.8 (12.8-304) | 29.6 (5.1-179) | 76.4 (13.2-392) | 54.6 (5.6-614) |
| LTB4, nM | 3.3 (0.8-25.4) | 2.6 (0.8-8.3) | 2.6 (0.6-22.9) | 3.1 (0.7-15.4) | 2.9 (0.7-8.9) |
| MMP-9, nM | 2.0 (0.1-45.1) | 2.6 (0.2-44.0) | 2.8 (0.0-30.9) | 4.8 (0.1-52.0) | 4.5 (0.0-58.3) |
| TIMP-1, nM | 5.3 (0.3-8.4) | 4.0 (1.6-10.5) | 5.4 (0.3-13.2) | 5.9 (0.5-16.3) | 3.7 (0.4-10.7) |
| Nitrite/nitrate, μM | 175 (86.0-525) | 161 (79.0-318) | 226 (73.0-1023) | 242 (61.0-1360) | 155 (67.0-1202) |
| NO SMOKING | | | | | |
| IL-8, pg/ml | 1766 (614-26524) | 1553 (462-8192) | 1216 (241-3478) | 1384 (379-12966) | 1628 (429-22476) |
| ECP, ng/ml | 72.1 (16.6-14040) | 75.1 (12.1-346) | 43.0 (15.5-346) | 46.3 (13.0-386) | 73.4 (8.0-804) |
| LTB4, nM | 4.1 (1.1-17.7) | 3.6 (1.0-14.4) | 2.6 (0.6-10.3) | 3.2 (0.7-13.4) | 3.4 (0.7-15.3) |
| MMP-9, nM | 3.3 (0.5-59.3) | 2.7 (0.1-75.1) | 1.6 (0.0-30.6) | 2.1 (0.0-69.1) | 1.2 (0.1-75.5) |
| TIMP-1, nM | 5.9 (0.6-10.8) | 5.5 (2.1-16.0) | 4.7 (2.2-14.3) | 4.6 (1.3-12.0) | 4.2 (0.8-14.4) |
| Nitrite/nitrate, μM | 150 (54.0-451) | 149 (66.4-403) | 169 (76.0-1324) | 254 (65.0-1279) | 150 (78.0-618) |

Values are expressed as medians (ranges). IL: interleukin, ECP: eosinophilic cationic protein, LTB4: leukotriene B4, MMP-9: matrix metalloproteinase-9, TIMP-1: tissue inhibitor of metalloproteinase-1,
